# Editorial: Advanced biocompatible piezoelectrics − synthesis, characterization, and applications

**DOI:** 10.3389/fbioe.2025.1739314

**Published:** 2025-11-13

**Authors:** Pavel Zelenovskii, Svitlana Kopyl, Joanna Bauer, Andrei Kholkin

**Affiliations:** 1 Department of Physics and CICECO – Aveiro Institute of Materials, University of Aveiro, Aveiro, Portugal; 2 Department of Biomedical Engineering, Faculty of Fundamental Problems of Technology, Wroclaw University of Science and Technology, Wroclaw, Poland; 3 Institute of Solid State Physics, University of Latvia, Riga, Latvia

**Keywords:** piezoelectrics, ferroelectrics, nanostructures, nanocomposites, biosensors, bioglass

Since its discovery, piezoelectricity has been a cornerstone of electromechanical energy conversion, yet its biomedical potential has remained largely unexploited. Traditional piezoelectrics, such as lead zirconate titanate (PZT) or barium titanate, though offering strong electromechanical coupling, are inherently rigid, brittle, and often toxic − poorly suited to the dynamic, compliant nature of biological tissues. On the other hand, synthetic polymers such as polyvinylidene difluoride (PVDF) and its copolymers provide flexibility but lack biodegradability and true biological integration.

In recent years, biocompatible piezoelectronics, the convergence of piezoelectricity and biocompatibility, has emerged as a new frontier in bioengineering and biotechnology. It merges materials science, nanotechnology and biomedical engineering to develop piezoelectric systems that not only efficiently convert mechanical stress into electrical signals but also coexist and function harmoniously within biological environments (Wang et al., 2022).

This Research Topic, “*Advanced Biocompatible Piezoelectrics − Synthesis, Characterization, and Applications”*, brings together five diverse yet thematically unified studies − two reviews and three original research articles − that collectively advance our understanding of how electromechanical phenomena can be harnessed for biomedical innovations. Together, they explore the interplay between structure, processing, and biological function, demonstrating both fundamental progress and practical potential.

The review *“Biodielectrics: old wine in a new bottle?”* by Barnana et al. serves as a conceptual cornerstone for this Research Topic. It reframes biological dielectric phenomena such as piezoelectricity, pyroelectricity, and ferroelectricity as intrinsic properties of hierarchically organized biological systems. From the polar organization of amino acids and peptides to the electromechanical behavior of collagen and cellulose, the review bridges molecular design with macroscopic functionality. The authors challenge researchers to think beyond synthetic analogs and toward biomimetic architectures − systems that not only replicate but also evolve the mechanisms of natural electromechanical transduction.

The mini-review *“Research progress of piezoelectric materials in protecting oral health and treating oral diseases”* by Yang et al. summarizes recent advances in the use of piezoelectric materials for oral disease treatment. The authors classify the piezoelectric materials and analyze therapeutic mechanisms, where locally generated electrical charges can regulate dentin tissue regeneration, antibacterial activity, mineralization, and periodontitis treatment. Importantly, the review identifies current limitations and bottlenecks in clinical translation of these materials and propose strategies for their real clinical applications in future.

The article by Yoneda et al. represents a milestone in healthcare applications of soft piezoelectrics. The authors utilized e-Rubber, an electroactive polymer-based artificial muscles, as a flexible piezo-capacitive pressure sensor to fabricate smart insoles for early detection of degenerative diseases such as osteoarthritis (Wipperman et al., 2024). By demonstrating low signal variability (below 8%) and sufficient precision for healthcare sensing, the study revealed the influence of environmental factors and mechanical housing on sensor accuracy. This work provides a strong example of how soft electromechanical materials can be validated for biomechanical sensing, rehabilitation monitoring, and pressure-distribution diagnostics − key steps toward wearable, real-time health monitoring systems.

The original study by Hammami et al. develops a novel biocompatible bioelectronic interface by doping 45S5 Bioglass® with conductive and bioactive additives. The addition of zirconium dioxide (ZrO_2_) and magnetite (Fe_3_O_4_) enhances both electrical conductivity and biological activity, with ZrO_2_ demonstrating particularly high performance. The improved conductivity and antibacterial properties are especially relevant for smart implant surfaces, where electrical stimulation can promote osseointegration and inhibit bacterial colonization (Miola et al., 2015). This work exemplifies how rational doping strategies can transform inert bioceramics into electrically responsive, biofunctional coatings, thus highlighting the potential of multifunctional composites in next-generation biomedical devices.

The contribution by Omar et al. demonstrates how microstructural control can yield flexible, responsive materials optimized for real biomedical signals. By tailoring electrospinning parameters and incorporating carbon black nucleating agents, the authors engineered a poly(vinylidene fluoride-co-hexafluoropropylene)-based nanocomposite exhibiting both capacitive and piezoelectric sensing. The material’s dual response to slow and fast pressure fluctuations makes it a promising candidate for multimodal physiological monitoring. Despite the synthetic origin of the composite, its modification toward higher β-phase crystallinity and biofunctionality bridges a gap between high-performance polymers and emerging biodegradable alternatives, moving toward more sustainable and biocompatible sensing materials.

Together, all these contributions define the trajectory of a rapidly evolving discipline − biocompatible piezoelectronics. From bioglass coatings to bioinspired dielectrics, and from smart insoles to oral regenerative systems, the scope of this Research Topic underscores a key principle: biocompatibility and functionality are no longer opposing design goals but mutually reinforcing ones.

Looking ahead, the next challenge is integration − creating systems-level piezoelectronic devices capable of real-time sensing, energy harvesting, and stimulation within living tissues, without compromising biological harmony. In a further perspective, a reshape of biomedical electronics from passive observation to active, intelligent interaction with the human body can be foreseen. Achieving these ambitious goals will require a deeper understanding of electromechanical coupling in soft, hydrated environments and the development of fabrication methods that respect both the fragility of life and the precision demands of advanced technology.

The Guest Editors of this Research Topic thank all contributing authors, reviewers, and the editorial office for their time and efforts in preparing, reviewing, and refining the manuscripts. We hope this collection will inspire researchers across disciplines and stimulate further discoveries in the field of biocompatible piezoelectronics.
